# Incorporating IL7 receptor alpha signaling in the endodomain of B7H3-targeting chimeric antigen receptor T cells mediates antitumor activity in glioblastoma

**DOI:** 10.1007/s00262-024-03685-7

**Published:** 2024-04-15

**Authors:** Nithidol Sakunrangsit, Nattarika Khuisangeam, Thananya Inthanachai, Varalee Yodsurang, Pasrawin Taechawattananant, Koramit Suppipat, Supannikar Tawinwung

**Affiliations:** 1 Department of Pharmacology and Physiology, Faculty of Pharmaceutical Sciences, Chulalongkorn University, Bangkok, 10330 Thailand; 2 Medical Microbiology, Interdisciplinary and International Program, Graduate School, Chulalongkorn University, Bangkok, 10330 Thailand; 3 Department of Biochemistry and Microbiology, Faculty of Pharmaceutical Sciences, Chulalongkorn University, Bangkok, 10330 Thailand; 4 Department of Research Affairs, Faculty of Medicine, Chulalongkorn University, Bangkok, 10330 Thailand; 5 Cellular Immunotherapy Research Unit, Chulalongkorn University, Bangkok, 10330 Thailand; 6 Thailand Hub of Talents in Cancer Immunotherapy (TTCI), Bangkok, 10330 Thailand

**Keywords:** Adoptive cell therapy, Chimeric antigen receptor, Interleukin-7 receptor, CD276, Glioblastoma

## Abstract

CAR-T-cell therapy has shown promise in treating hematological malignancies but faces challenges in treating solid tumors due to impaired T-cell function in the tumor microenvironment. To provide optimal T-cell activation, we developed a B7 homolog 3 protein (B7H3)-targeting CAR construct consisting of three activation signals: CD3ζ (signal 1), 41BB (signal 2), and the interleukin 7 receptor alpha (IL7Rα) cytoplasmic domain (signal 3). We generated B7H3 CAR-T cells with different lengths of the IL7Rα cytoplasmic domain, including the full length (IL7R-L), intermediate length (IL7R-M), and short length (IL7R-S) domains, and evaluated their functionality in vitro and in vivo. All the B7H3-IL7Rα CAR-T cells exhibited a less differentiated phenotype and effectively eliminated B7H3-positive glioblastoma in vitro. Superiority was found in B7H3 CAR-T cells contained the short length of the IL7Rα cytoplasmic domain. Integration of the IL7R-S cytoplasmic domain maintained pSTAT5 activation and increased T-cell proliferation while reducing activation-induced cell death. Moreover, RNA-sequencing analysis of B7H3-IL7R-S CAR-T cells after coculture with a glioblastoma cell line revealed downregulation of proapoptotic genes and upregulation of genes associated with T-cell proliferation compared with those in 2nd generation B7H3 CAR-T cells. In animal models, compared with conventional CAR-T cells, B7H3-IL7R-S CAR-T cells suppressed tumor growth and prolonged overall survival. Our study demonstrated the therapeutic potential of IL7Rα-incorporating CAR-T cells for glioblastoma treatment, suggesting a promising strategy for augmenting the effectiveness of CAR-T cell therapy.

**Supplementary Information:**

The online version contains supplementary material available at 10.1007/s00262-024-03685-7.

## Introduction

Chimeric antigen receptor (CAR) T cell therapy has shown remarkable success in treating hematologic malignancies and has been approved by the FDA for CD19-targeted and B-cell maturation antigen (BCMA)-targeted CARs [1, 2]. A recent study further supported the notion that CAR-T cell therapy holds promise as a potential therapeutic option for solid tumors [3]. However, the clinical efficacy of CAR-T cell therapy for solid tumors is limited [4, 5] due to the suppressive tumor microenvironment that renders T cell dysfunction and poor persistence [6, 7]. To address this limitation, new strategies for enhancing adoptive cell therapy in solid tumors are urgently needed.

Optimal T-cell activation and durable antitumor efficacy require three signals: TCR activation (signal 1), costimulation (signal 2), and immunostimulatory cytokines (signal 3) [8]. One way to achieve this goal is by engineering 2nd generation CAR-T cells that are equipped with immunostimulatory cytokines. Interleukin-7 (IL7) profoundly affects T-cell activation, differentiation, and homeostasis, and IL7 has been shown to maintain the survival and proliferative capacity of naïve T cells [9–11]), suggesting a potential therapeutic benefit. Recent studies have demonstrated that genetically modified T cells that constitutively express IL7 signaling components, such as secreted IL7 [12–14], or constitutively express the IL7 receptor alpha as a signal 3 can improve their antitumor efficacy [15, 16].

B7H3 (CD276) is a type I transmembrane glycoprotein and a member of the B7 costimulatory molecule family [17, 18]. Previous studies have shown high expression levels of B7H3 on the cell surface in various solid tumors, such as glioblastoma [19, 20], breast cancer [21], cholangiocarcinoma [22], pancreatic cancer [20, 23], and colorectal cancer [24], but low expression in normal human tissue [25]. High expression of B7H3 has been closely correlated with poor prognosis and inferior clinical outcomes [23, 26, 27]. B7H3 CAR-T cell therapy has been utilized to treat a variety of solid tumors, including neuroblastoma, glioblastoma, atypical teratoma/rhabdomyoma, and Ewing’s sarcoma [19, 28, 29].

In this study, we developed novel B7H3-targeting CAR constructs that incorporated the intracellular signaling domain of IL7Rα in the endosignaling domain. We investigated the antitumor capacity of these CAR constructs in vitro and in vivo. Our results demonstrated that these intrinsic IL7Rα-CAR-T cells exhibit enhanced antitumor activity and T-cell persistence against glioblastoma compared with conventional 2nd generation B7H3 CAR-T cells. This study provides a promising new approach to enhancing the efficacy of CAR-T cell therapy against solid tumors, with potential implications for clinical translation.

## Materials and methods

### Cell lines and cultures

The human glioblastoma cell lines (LN229 and U87), a B-cell acute lymphoblastic leukemia cell line (NALM-6) and the human embryonic kidney cell line 293 (293T) used in this study were purchased from ATCC (Manassas, USA) and maintained at a low passage number. The cell lines were cultured in Dulbecco’s modified Eagle’s medium (DMEM) or Roswell Park Memorial Institute (RPMI)-1640 medium (Gibco, USA) supplemented with 10% fetal bovine serum (FBS) (Gibco, USA), 1% GlutaMax (Invitrogen, USA), and 1% penicillin‒streptomycin (Corning, USA) at 37 °C in a 5% CO_2_ incubator and were routinely tested for Mycoplasma contamination. To detach adherent cells from the plastic culture flasks, 0.25% trypsin-0.02% EDTA solution (Invitrogen, USA) was utilized.

### Isolation and activation of primary human T lymphocytes

PBMCs were isolated from the peripheral blood of healthy donors by gradient centrifugation using a Ficoll histopaque gradient method (Sigma‒Aldrich, USA). The PBMCs were washed with 1X PBS (Gibco, USA) and resuspended in TexMACS medium (Miltenyi Biotec, Germany) supplemented with 5% FBS to a density of 2 × 10^6^ cells/well in 24-well plates. T cells were activated using T-Cell TransAct™ (Miltenyi Biotec, Germany) supplemented with 50 U/ml human IL-2 (Miltenyi Biotec, Germany) for 3 days before introducing the viral vector for CAR-T cell generation.

### Generation of CAR -T cells

The B7H3-CAR constructs contain a signal peptide of Ig Heavy chain, a B7H3-specific single-chain variable fragment (scFv), an IgG2-derived CH3 hinge, and an endodomain composed of the following intracellular signaling components: 41BB (UniProtKB: Q07011), various lengths of IL7Rα (UniProtKB: P16871) and CD3ζ (UniProtKB: P20963). Detailed sequences of B7H3-CAR constructs are available upon request (email should be addressed to ST). Gamma (γ)-retroviruses were generated by transfecting 293T cells with the plasmid encoding the B7H3-CAR construct, the plasmid containing the MomLV gag-pol sequence, and the RD114 envelope plasmid using GeneJuice® Transfection Reagent (Sigma‒Aldrich, USA) according to the manufacturer’s instructions. Supernatants were collected at 48 and 72 h, filtered with 0.45 μm Supor® Membrane (Pall, USA), quickly frozen in dry ice-ethanol baths, and preserved at − 80 °C until use.

The viral titer of each B7H3-CAR construct was determined by measuring the expression of the CAR in 293T cells through flow cytometry. Briefly, 5 × 10^4^ 293T cells were seeded overnight in 24-well plates before transduction. The γ-retroviral supernatant was thawed and added to 293T cells by serial dilution from 10^0^ to 10^–4^ in complete DMEM supplemented with 8 μg/ml polybrene (Sigma‒Aldrich, USA). One day later, the medium containing the virus was discarded, and the medium was replaced with fresh complete DMEM. The cells were collected and stained for CAR-T-cell expression after 3 days of titration. The number of transduction units per mL (TU/ml) of each construct was calculated using the following formula:

$${\text{TU}}/{\text{ml }} = \frac{{{\text{Frequency of CAR }} \times 50,000{\text{ cells }} \times {\text{ dilution factor }}}}{{0.5{\text{ ml}}}}$$

The amount of virus needed to achieve a multiplicity of infection (MOI) of 2 was calculated based on the TU/ml and the initial number of T cells. Nontissue culture-treated 24-well plates were coated with 7 µg/mL RetroNectin® (Takara Bio, Inc., Japan) in PBS at 4 °C overnight. After removal of the coating mixture, the retroviral supernatant of each CAR construct was added at an MOI of 2 and centrifuged at 2000 × g for 90 min. The virus was gently discarded before seeding the well with 2 × 10^5^ activated T cells in complete TexMAC medium supplemented with 10 ng/ml human IL-7 (200 U/mL) (Miltenyi Biotec, Germany) and 5 ng/ml human IL-15 (10 U/mL) (Miltenyi Biotec, Germany) for transduction. The plates were centrifuged at 400 × g for 5 min. Then, the cells were continuously cultured for 11–18 days in complete TexMAC medium supplemented with 10 ng/ml human IL-7 and 5 ng/ml human IL-15, and the cells were collected for in vitro and in vivo studies.

The expression levels of the anti-B7H3 CARs were detected via flow cytometry with a human B7H3 protein, an Fc tag (Cat# B73-H5253, Acro, USA) and an allophycocyanin (APC) anti-Fc tag (Biolegend, CA, USA).

### Flow cytometry and antibodies

Antibodies used in flow cytometry were as follows: anti-human B7H3 (Polyclonal Goat IgG, Cat# AF1027, R&D systems), anti-Goat IgG (H + L) (polyclonal donkey IgG, Cat# F0107, R&D systems), PE anti-human CD3 (clone OKT3, Cat# 317,308, Biolegend, USA), PerCP anti-human CD3 (clone OKT3, Cat# 317,336, Biolegend, USA), APC anti-human CD3 (clone OKT3, Cat# 317,318, Biolegend, USA), APC anti-human CD4 (clone OKT4, CAT# 317,416, Biolegend, USA), PE anti-human CD8 (clone SK1, Cat#344,706, Biolegend, USA), FITC anti-human CD8 (clone SK1, Cat# 344,704, Biolegend, USA), Alexa Fluor 488 anti-Stat5 (clone 47/Stat5(pY694), Cat# 612,598, BD Bioscience, USA), APC anti-human CD45RO (clone UCHL1, Cat#304,210, Biolegend, USA), FITC anti-human CD62L (clone DREG-56, Cat#304,804, Biolegend, USA), APC-Cy7 anti-human CD69 (clone FN50, Cat# 310,910, Biolegend, USA), PE anti-human TIGIT (clone A151536, Cat# 372,704, Biolegend, USA), APC anti-human TIM-3 (clone F38-2E2, Cat# 345,012, Biolegend, USA), and FITC anti-human PD-1 (clone EH12.2H7, Cat# 329,904, Biolegend, USA). Flow cytometry was performed using a BD Accuri™ C6 Plus flow cytometer (BD Biosciences, USA), and the data were analyzed using FlowJo software v.10 (TreeStar, USA).

### Cytotoxicity assay

Tumor cells in complete DMEM were seeded and allowed to attach to 24-well plates (Corning, USA) overnight. Then, different effector cells were added to the target cells (at an E:T ratio) at 1:1, 1:2 or 1:4. After 72 h, the cocultured cells were harvested and resuspended in an identical volume of staining buffer. 7-AAD dye was added to exclude dead cells. The number of viable target cells and effector cells was counted using an Accuri C6 flow cytometer with a fixed acquisition volume [30]. Specific lysis was calculated using the following formula:

$${\text{Specific lysis }}\left( {\text{\% }} \right) = { }100 - \left( {\frac{{{\text{Number of target cell after co}} - {\text{cultured}}}}{{\text{Number of target cell in target alone conditon}}} \times 100} \right)$$

### T-cell activation and apoptosis assays

To analyze the biological characteristics of the CAR-T cells, effector cells were cultured with targets at a 1:1 ratio in a 24-well plate without the addition of exogenous cytokines for 72 h. The cells were harvested and analyzed for the expression of CD25 and CD69 [31]. Additionally, Annexin V and 7-AAD were combined to analyze the apoptosis of T cells.

### Intracellular STAT5 phosphorylation

Effector cells were harvested into FACS tubes and cultured with target cells at a 2:1 (E:T) ratio in complete medium without additional cytokines. Thirty to 60 min later, the cells were washed in cold 1 × PBS containing 5% FBS (flow buffer) and centrifuged at 250 × g for 5 min. The cells were fixed with fixative buffer at 37 °C for 10 min before slowly adding ice-cold Perm buffer II. After permeabilization at 4 °C for 30 min, the cells were centrifuged, the supernatant was discarded, and the cells were washed with cold flow buffer. Then, the cells were stained with 5 μL of anti-human pSTAT5 and 20 μL of anti-human CD3 antibodies at room temperature in the dark. After 30 min, the cells were washed with cold flow buffer and then immediately analyzed through flow cytometry.

### Cytokine analysis

For cytokine measurements, effector cells were cocultured with tumor cells at a 1:1 ratio without the addition of exogenous cytokines. After 24 h, the supernatant was collected, and cytokine secretion was assessed using a BD cytometric bead array (CBA) (BD Biosciences, USA) according to the manufacturer’s instructions.

### RNA isolation and RNA sequencing

After 72 h of coculture, CAR-T cells were collected and enriched with an EasySep™ human CD3 + selection kit II (STEMCELL Technologies) following the manufacturer’s instructions. Total RNA was extracted from CD3^+^ T cells using an RNeasy Mini Kit (QIAGEN) with DNase to remove the DNA. RNA quality was assessed using a Nanodrop One spectrophotometer (Thermo Fisher Scientific). The RNA integrity was evaluated from 18 and 28S ribosomal RNA bands and the 28S/18S ratio using 1% agarose gel electrophoresis and Quantity One (Gel Doc XR, Bio-Rad, USA). The RNA integrity number (RIN) was measured using an Agilent 2100 Bioanalyzer (Agilent Technologies). A 2 µg total RNA sample with a RIN > 7 was used as a template for stranded RNA-seq library construction following the manufacturer’s instructions. Illumina NovaSeq 150-bp paired-end sequencing was performed by Vishuo Biomedical (Thailand) Ltd.

### Differential expression analysis

RNA samples were subjected to RNA-seq analysis. The quality of the raw sequence read data in FASTQ files was assessed using the FastQC tool. Clean reads that passed the quality filter were trimmed using Cutadapt (version 4.2) and aligned to the reference human transcriptome from Ensembl (version 96) using Kallisto (version 0.45.1) [32]. Differential gene expression analysis of the samples was performed using EdgeR with thresholds of a duplicate CPM > 0.5 and an FDR < 0.5. Log2-fold changes of < 0.5 and > 2.0 were defined as significant downregulation and upregulation, respectively.

For data visualization, heatmaps were generated using the R Bioconductor package. The Database for Annotation, Visualization and Integrated Discovery (DAVID) tool was used to identify the Gene Ontology (GO) terms related to the biological processes and molecular functions of the selected significant differentially expressed genes (DEGs).

### Xenograft mouse models

Six-week-old female NOD/ShiJic-scid Jcl (NOD-SCID) mice were obtained from Nomura Siam International (Bangkok, Thailand). At eight weeks of age, the mice were engrafted subcutaneously (s.c.) into the right flank with a 1:1 mixture of 5 × 10^6^ tumor cells (LN229 or U87) and Matrigel matrix (Corning, USA). When the tumor volumes were between 50 and 70 mm^3^, the mice were randomly divided into 3 groups and treated with 1 × 10^7^ nontransduced, B7H3- or B7H3-IL7R-S CAR-T cells intravenously (i.v.) for 2 doses at 3-day intervals. Body weight, tumor size, and the onset of GVHD were monitored weekly. All efforts were made to minimize animal suffering. Mice were euthanized when tumor ulceration and/or tumor volume reached 15 mm in diameter. Tumor volume was calculated using the following formula:

$${\text{Tumor volume }} = { }\frac{{{\text{length }} \times {\text{weight}}^{2} }}{2}$$

### Statistical analysis

GraphPad Prism software v.7 (GraphPad, San Diego, CA, USA) was used to analyze and visualize the results. One-way or two-way ANOVA followed by Tukey’s multiple comparison test was performed to assess differences between groups or between each group and the indicated control. The sample size for the animal experiments was calculated using computerized statistical power analyses (G*Power program). The input parameters for the analysis were as follows: Statistical test = One-way ANOVA, Type of power analysis = A priori: computed required sample size, Effect size (f) = 1.3, Alpha error probability = 0.05, Power (1-Beta error probability) = 0.8, Number of groups = 3. Therefore, a minimum of 4 mice in each group is needed for the study. Survival determined from the time of tumor cell injection was analyzed by the Kaplan–Meier method, and differences in survival between groups were compared by the log-rank test. The data are shown as the mean ± SEM. *P < 0.05; **P < 0.01; ***P < 0.001, significant; ns, not significant.

## Results

### Generation of novel B7H3-targeting CARs with an intracellular signaling domain of IL7Rα

**Fig. 1:**
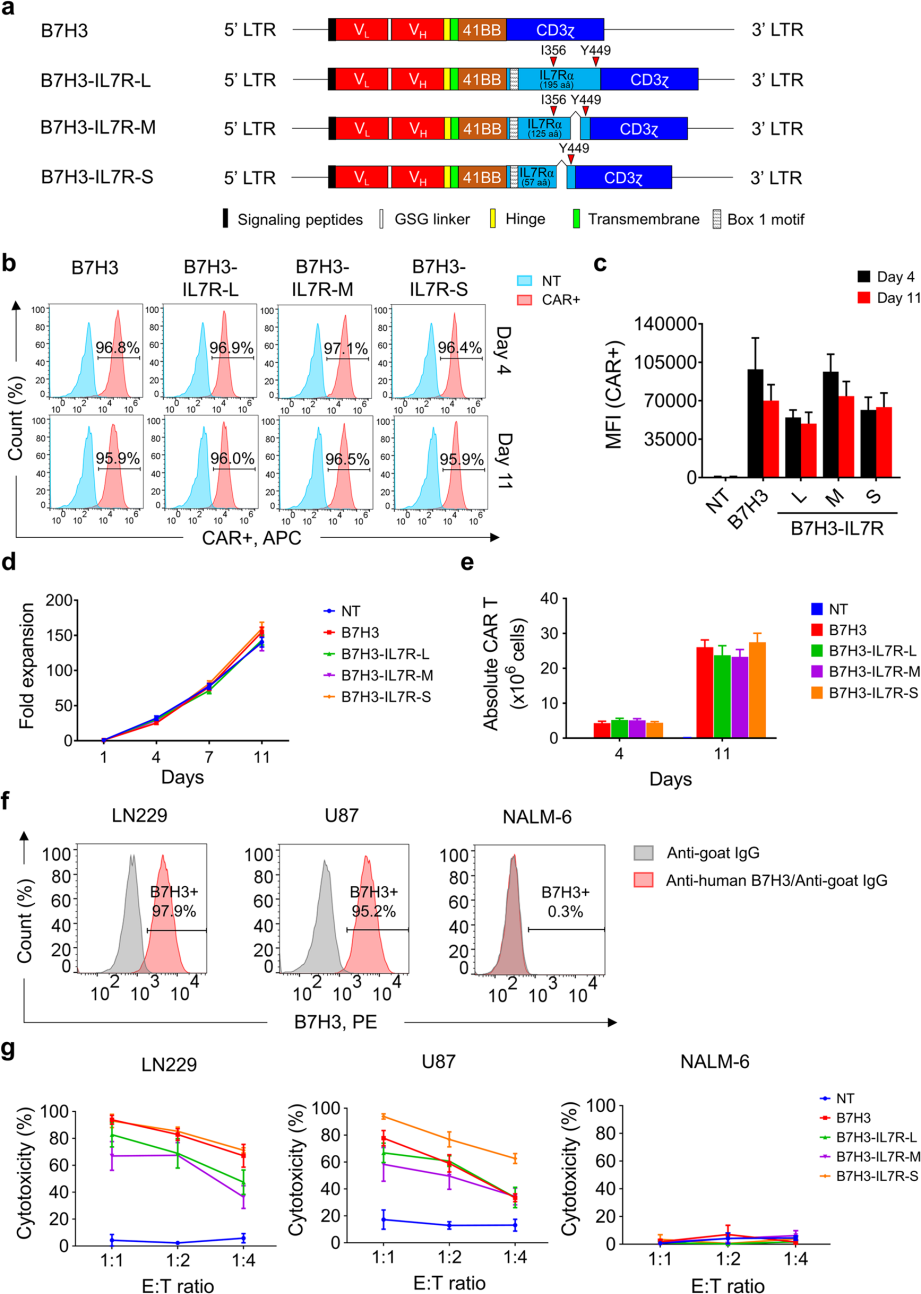
Generation of B7H3-targeting CAR-T cells harboring the cytoplasmic domain of IL7Rα. **a** Schematic of B7H3 CAR constructs with different lengths of IL7Rα. **b** Histogram displaying the expression of B7H3-specific CARs in transduced activated T lymphocytes. **c** Bar graph representing the mean fluorescence intensity (MFI) at 4 and 11 days after transduction. **d** Fold expansion of nontransduced cells and of each type of CAR-T cell at 1, 4, 7 and 11 days after transduction. **e** Absolute numbers of CAR^+^ cells at days 7 and 11. **f** B7H3 expression in GBM (LN229 and U87) and B-cell acute lymphoblastic leukemia (NALM-6) cell lines screened by flow cytometry using a goat anti-human B7H3 polyclonal antibody as the primary antibody and PE-conjugated anti-goat IgG as the secondary antibody. **g** Cytotoxic activity of CAR-T cells against the LN229, U87 and NALM-6 cell lines after 72 h of coculture at E:T ratios of 1:1, 1:2 and 1:4. The data are presented as the mean ± S.E.M. (*n* = 4)

We generated novel B7H3-specific CARs with an intracellular signaling domain of IL7Rα to provide additional cytokine signals to CAR-T cells. To accomplish this, we inserted the full-length or truncated intracellular domain of IL7Rα between the cytoplasmic domain of 41BB and CD3ζ, as described in Fig. 1a. B7H3-IL7R-L consists of the full-length intracellular domain of endogenous IL7Rα. The intermediate length (IL7R-M) contains the truncated intracellular domain of IL7Rα proximal to the membrane, up to amino acid position 360, and the Y449 C-terminal region, and the short length B7H3-IL7R-S consists of the Box1 motif and Y449 tyrosine residues derived from the cytoplasmic domain of IL7Rα. A second-generation B7H3 CAR construct containing only the intracellular domain of 41BB and CD3ζ (B7H3) was also generated as a control. We transduced each B7H3 CAR into activated T cells using a retroviral vector. The transduction efficiency of B7H3 CAR-T cells containing different lengths of the IL7Rα cytoplasmic domain was comparable to that of conventional B7H3 CAR-T cells (Fig. 1b − c). After T-cell culture, there were no significant differences in the fold expansion or the number of CAR^+^ cells among the CAR constructs (Fig. 1d − e). We evaluated the cytotoxic effects of CAR-T cells on B7H3^+^ glioblastoma (GBM) cell lines, namely, LN229 and U87. B7H3 and B7H3-IL7Rα CAR-T cells lysed B7H3-expressing cells in a dose-dependent manner and demonstrated antigen-specific killing, as they did not kill B7H3- target or Nalm-6 cells (Fig. 1f − g).

### Immune characteristics and antigenic responses of B7H3 CAR-T cells with an intracellular signaling domain of IL7Rα

**Fig. 2:**
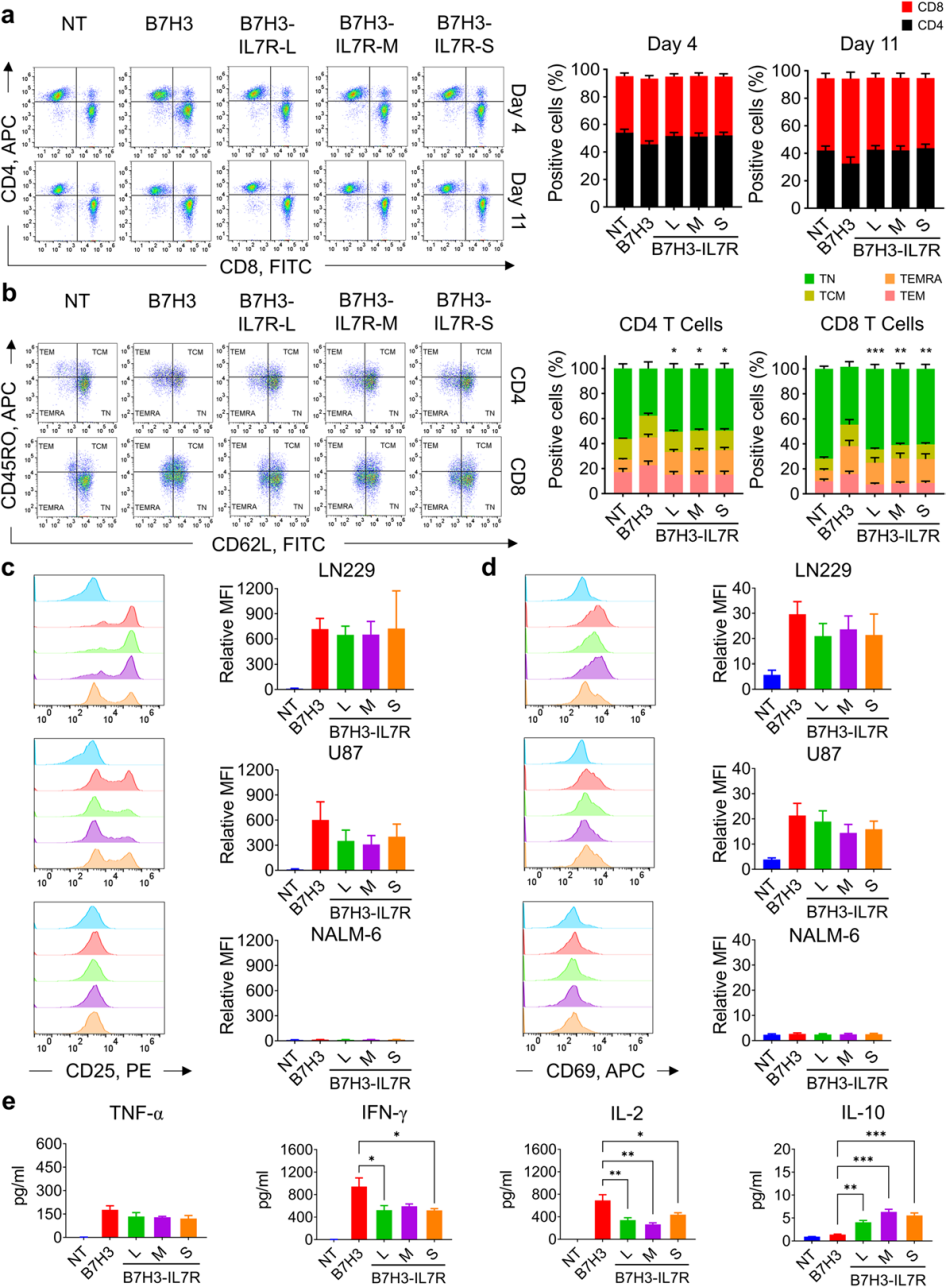
Immune characteristics and antigenic responses of B7H3 CAR-T cells to the intracellular signaling domain of IL7Rα. **a** CD4^+^ and CD8^+^ T-cell subsets in transduced cells at days 4 and 11. **b** Flow cytometry analysis of the memory T-cell subpopulations identified as follows: naïve T-cell (TN, CD45RO-CD62L+), central memory T-cell (TCM, CD45RO+CD62L+), effector memory T-cell (TEM, CD45RO + CD62L-), and effector T-cell (TEMRA, CD45RO-CD62L-) subsets in CAR-T cell products on day 11 after transduction. The data are shown as the mean ± S.E.M. (*n* = 4) ^*^P < 0.05, ^**^P < 0.01, ^***^P < 0.001 versus B7H3 (two-way ANOVA). **c** Expression of the activation markers CD25 and **d** CD69 on CAR-T cells after coculture with LN229, U87, or Nalm-6 cells for 72 h. The bar graph shows the relative MFI based on the isotype control. The data are shown as the mean ± S.E.M. (*n* = 4). **e** Cytokine secretion by CAR-T cells stimulated with LN229 cells at a 1:1 ratio for 24 h. The data are shown as the mean ± S.E.M. (*n* = 3) ^*^P < 0.05, ^**^P < 0.01, ^***^P < 0.001 (two-way ANOVA)

We next proceeded to evaluate the T-cell subsets and memory phenotypes of the different constructs on day 11 post-transduction. There were no differences in CD4 and CD8 ratios between the constructs (Fig. 2a). To characterize T-cell memory phenotypes, we employed a combination of the CD45RO and CD62L markers. The memory phenotype of the constructs containing IL7Rα was comparable to that of the NT cells, with the majority consisting of naïve T cells (CD45RO^−^CD62L^+^). However, in conventional 2nd generation B7H3 CAR-T cells, we observed a significantly lower population of naïve T cells compared to NT and IL7Rα CAR-T cells (Fig. 2b). When CD45RA and CCR7 were employed to characterize the memory phenotype, our results were consistent. We observed a greater proportion of naïve phenotype in CD8^+^ population of B7H3-IL7R CAR-T cells comparing with conventional B7H3 CAR-T cells (Fig. S1). Next, we assessed the antigenic responses of B7H3-IL7Rα CAR-T cells. All CAR-T-cell groups exhibited upregulation of CD69 and CD25 upon antigen stimulation, although no significant differences were observed among the groups (Fig. 2c − d). The cytokine secretion of B7H3 CAR-T cells after stimulation with the target antigen for 24 h was also evaluated. When encountering B7H3-positive targets, all CAR-T cells secreted various cytokines, including IL-2, IFN-γ, TNF-α, and IL-10. Interestingly, we observed that B7H3-IL7Rα CAR-T cells exhibited lower levels of IFN-γ and IL-2 than did conventional B7H3 CAR-T cells. In contrast, these cells secreted more IL-10 than did B7H3 CAR-T cells (Fig. 2e).

### Incorporation of IL7Rα signaling maintains phospho-STAT5 expression after antigen stimulation

**Fig. 3:**
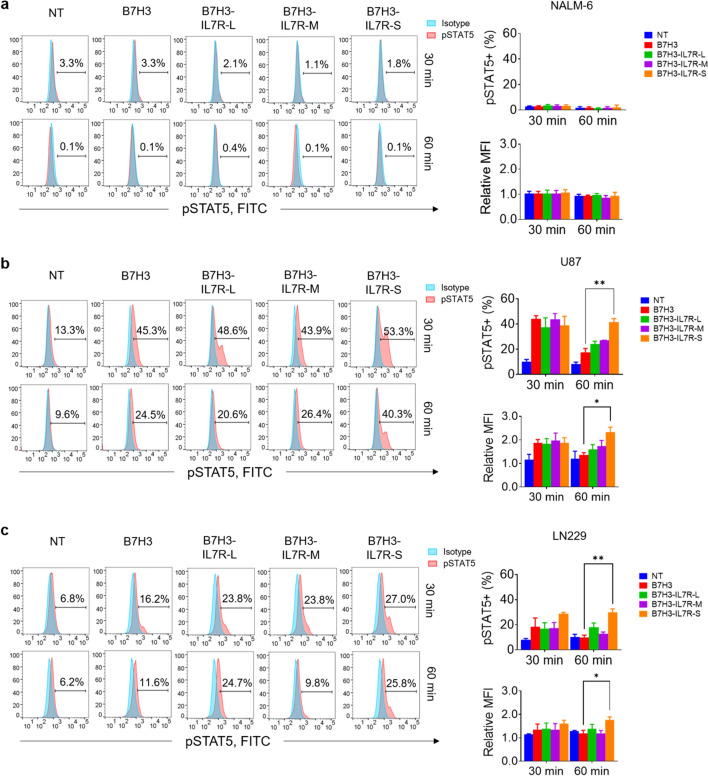
Incorporation of the IL7Rα signaling domain maintains phospho-STAT5 expression after antigen stimulation. **a** Flow cytometric analysis of intracellular pSTAT5 expression in B7H3-targeting CAR-T cells after stimulation with NALM-6 for 30 and 60 min. Histograms of pSTAT5 expression in the CD3^+^ population were generated. The percentage of pSTAT5^+^ T cells and the relative MFI of pSTAT5 were analyzed and are shown in the bar graphs. **b** Flow cytometric analysis of intracellular pSTAT5 expression in CAR-T cells stimulated with U87 cells or **c** LN229 cells for 30 or 60 min. The data are presented as the mean ± S.E.M. (*n* = 4) ^*^P < 0.05, ^**^P < 0.01, ^***^P < 0.001 (two-way ANOVA)

Furthermore, we investigated STAT5 phosphorylation in various B7H3 CAR-T cells after stimulation with the B7H3-positive glioblastoma (GBM) cell lines U87 and LN229. Upon stimulation with the B7H3-negative cell line (NALM-6), all CAR-T cells did not activate pSTAT5 in any of the constructs (Fig. 3a), whereas upon stimulation with B7H3^+^ target cells, all CAR-T cells demonstrated upregulation of pSTAT5 in an antigen-dependent manner (Fig. 3b–c). At 30 min after stimulation, we did not observe differences in pSTAT5 expression between the second-generation B7H3 CAR-T cells and the B7H3 CAR-T cells with additional IL7Rα signals. Interestingly, the B7H3-IL7R-S construct sustained pSTAT5 expression after 60 min of antigen exposure, with levels significantly higher than those observed in B7H3 CAR-T cells. Similar results were obtained when the cells were stimulated with either LN229 or U87 cells.

### B7H3 CAR-T cells composed of short-length IL7Rα (B7H3-IL7R-S) exhibit superior antitumor activity in a tumor rechallenge assay

**Fig. 4:**
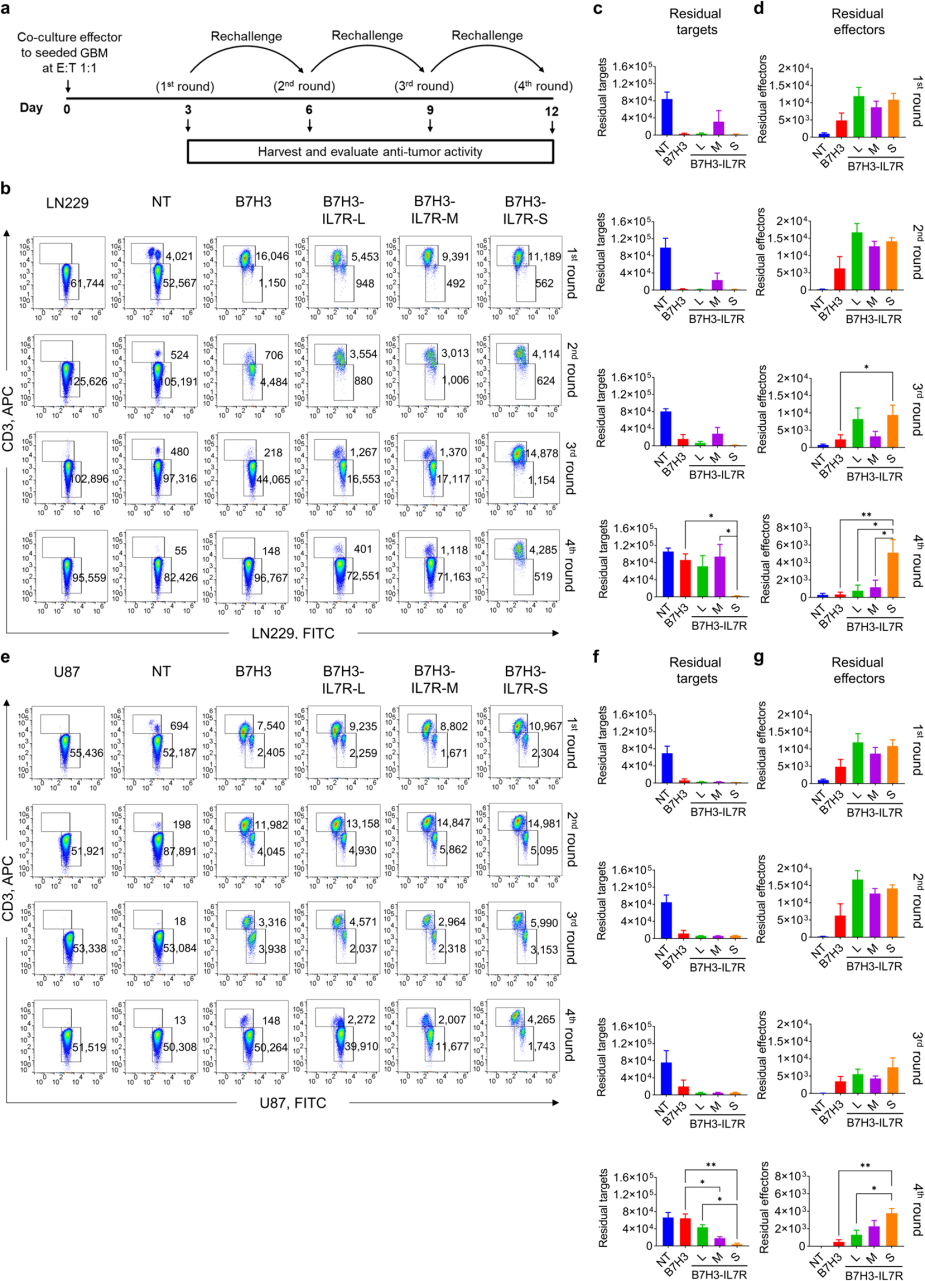
In vitro antitumor activity of B7H3-IL7Rα CAR-T cells in the tumor rechallenge experiment. **a** Schematic representation of the in vitro tumor rechallenge assay. B7H3-CAR-T cells were cocultured with GBM tumor cells (LN229 or U87) at a 1:1 E:T ratio. After 72 h, the cultures were harvested and analyzed for residual target cells and effector cells. The residual T cells were then cultured with new tumor cells for three additional rounds. **b** Representative flow cytometric analysis of the rechallenge assay using LN229 cells. **c** The residual target cells from each round of coculture were counted by an Accuri C6 flow cytometer with a fixed acquisition volume. **d** The remaining effector cells from each round of coculture. The results are presented as the mean ± S.E.M. (*n* = 4) ^*^P < 0.05, ^**^P < 0.01 (one-way ANOVA). **e** Representative flow cytometric analysis of U87 cells in the rechallenge assay. **f** The residual target cells from each round of coculture. **g** The remaining effector cells from each round of coculture. The results are presented as the mean ± S.E.M. (n = 3). ^*^P < 0.05, ^**^P < 0.01 (one-way ANOVA)

Next, we further explored whether B7H3-IL7Rα CAR-T cells had functional advantages in terms of antitumor activity and T-cell proliferation over conventional B7H3 CAR-T cells using an in vitro tumor-rechallenge assay. Figure 4a shows the experimental scheme of the rechallenge assay, in which the remaining CAR-T cells were collected and recultured with new tumor cells for a total of four rounds. The experiments were conducted using two different GBM cell lines, LN229 (Fig. 4b–d) and U87 (Fig. 4e–g). During the initial round of coculture, B7H3 CAR-T cells from all the groups effectively eliminated both GBM cell types. However, B7H3, B7H3-IL7R-M and B7H3-IL7R-L CAR-T cells exhibited a reduction in their cytotoxic function toward both target cells after the fourth exposure. In contrast, B7H3-IL7R-S CAR-T cells maintained their cytotoxic function against GBM cells (Fig. 4c, f). Moreover, B7H3-IL7R-S CAR-T cells demonstrated a significantly higher number of effector cells than did conventional B7H3 CAR-T cells after the fourth re-exposure to the GBM cells (Fig. 4d, g). Analysis of the phenotypes of CAR-T cells at the end of the third round of tumor rechallenge was also performed. The majority of the remaining CAR-T cells were effector memory cells, and there were no significant differences in the memory phenotype among the B7H3 CAR-T groups. Notably, compared with conventional B7H3 CAR-T cells, B7H3-IL7R-S cells exhibited lower PD1 and LAG-3 upregulation (Fig. S2). These findings suggested that the incorporation of a short length of IL7Rα (IL7R-S) results in better antitumor activity and T-cell persistence after repeated antigen exposure than the incorporation of other constructs.

### B7H3-IL7R-S CAR-T cells exhibited distinct transcription profiles

**Fig. 5:**
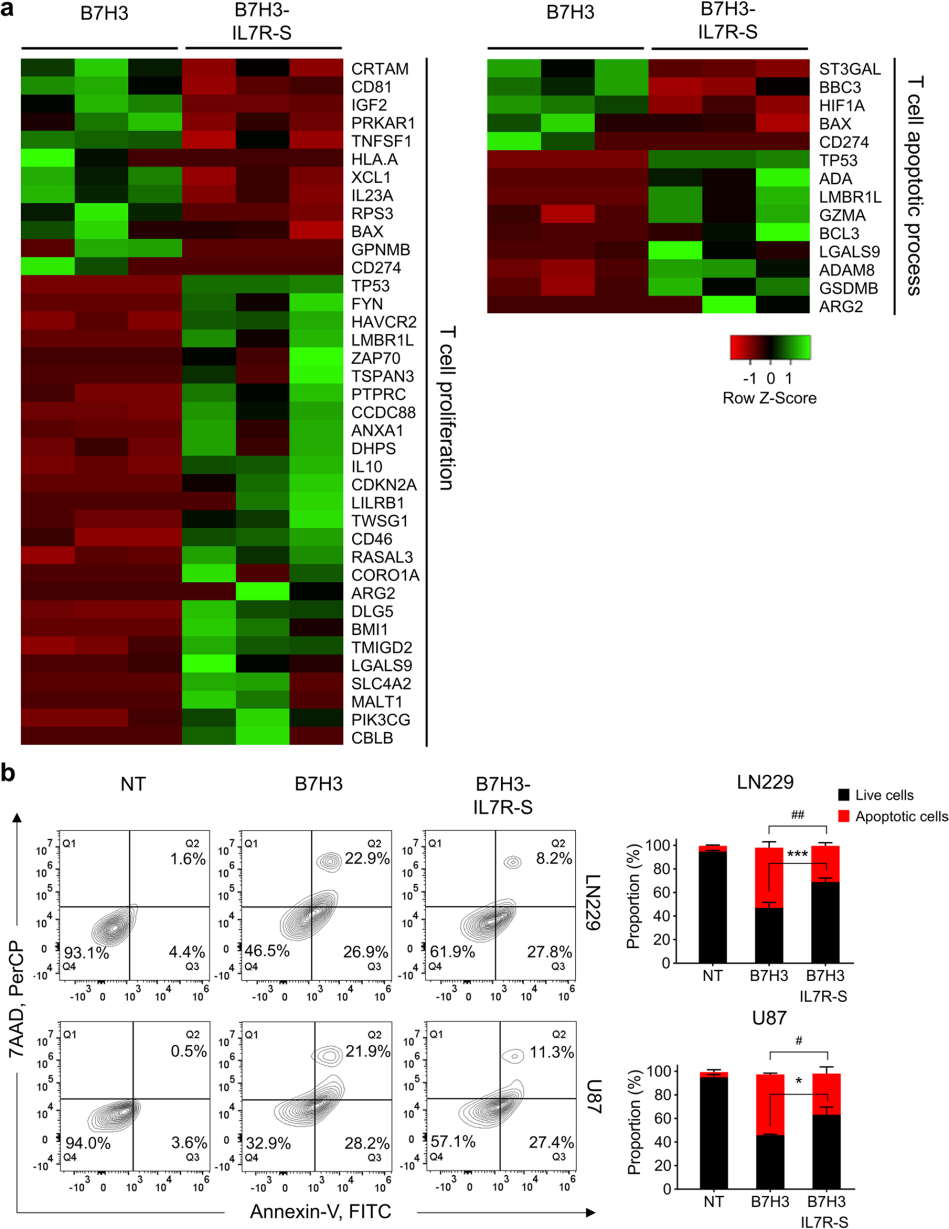
B7H3-IL7R-S CAR-T cells exhibited distinct transcription profiles and decreased apoptosis induction**. a** Heatmap illustrating the relative gene expression associated with T-cell proliferation and T-cell apoptotic process in CAR T cell stimulated with B7H3^+^ target from three donors. Red indicates downregulated mRNA abundance, while green indicates upregulated mRNA abundance of selected significant genes with a fold change (FC) either below 0.5 or above 2. **b** Flow cytometric plot of programmed cell death in CAR-T cells after coculture with LN229 or U87 cells for 72 h. Apoptotic cells were gated from CD3^+^ cells, and the percentages of live and apoptotic cells are shown in the bar graph. The data are presented as the mean ± SEM. (*n* = 4) ^*^P < 0.05, ^**^P < 0.01 for live cells compared with B7H3 CAR-T cells; ^#^*P* < 0.05, ^##^*P* < 0.01 for apoptotic cells compared with B7H3 CAR-T cells

To gain further insights into the molecular pathway underlying the enhanced functionality of B7H3-IL7R-S CAR-T cells, we performed RNA sequencing (RNA-seq) analysis on 2nd generation B7H3 and B7H3-IL7R-S CAR-T cells that were cocultured with B7H3^+^ target cells. After 72 h of coculture, the CAR-T cells were harvested and enriched using a CD3^+^ selection kit, after which RNA sequencing was subsequently performed. We compared the DEGs between conventional B7H3 CAR-T cells and B7H3-IL7R-S CAR-T cells. Gene Ontology (GO) analysis was performed to identify hub genes involved in T-cell proliferation and T-cell apoptosis. A heatmap analysis revealed greater expression of transcriptional regulators associated with T-cell proliferation, such as *FYN*, *ZAP70*, *PTPRC*, *IL10*, *TWSG1*, and *CD46,* in B7H3-IL7R-S cells than in B7H3 CAR-T cells. Conversely, genes related to apoptotic processes and programmed cell death, including *ST3GAL*, *BAX*, *BBC3*, and *CD274*, were downregulated in B7H3-IL7R-S CAR-T cells compared to conventional B7H3 CAR-T cells. We also observed significant upregulation of antiapoptotic genes such as *BCL3* and *ADAM8* in B7H3-IL7R-S CAR-T cells **(**Fig. 5a**)**. Furthermore, we investigated whether the incorporation of a shorter IL7Rα signaling domain reduced activation-induced cell death in B7H3 CAR-T cells. As shown in Fig. 5b, B7H3-IL7R-S cells exhibited lower apoptosis levels than did conventional second-generation B7H3 CAR-T cells when cocultured with LN229 and U87 cells, supporting the downregulation of genes associated with apoptosis observed via GO analysis.

### B7H3-IL7R-S CAR-T cells eliminate tumor progression and prolong survival in xenograft GBM mouse models

**Fig. 6:**
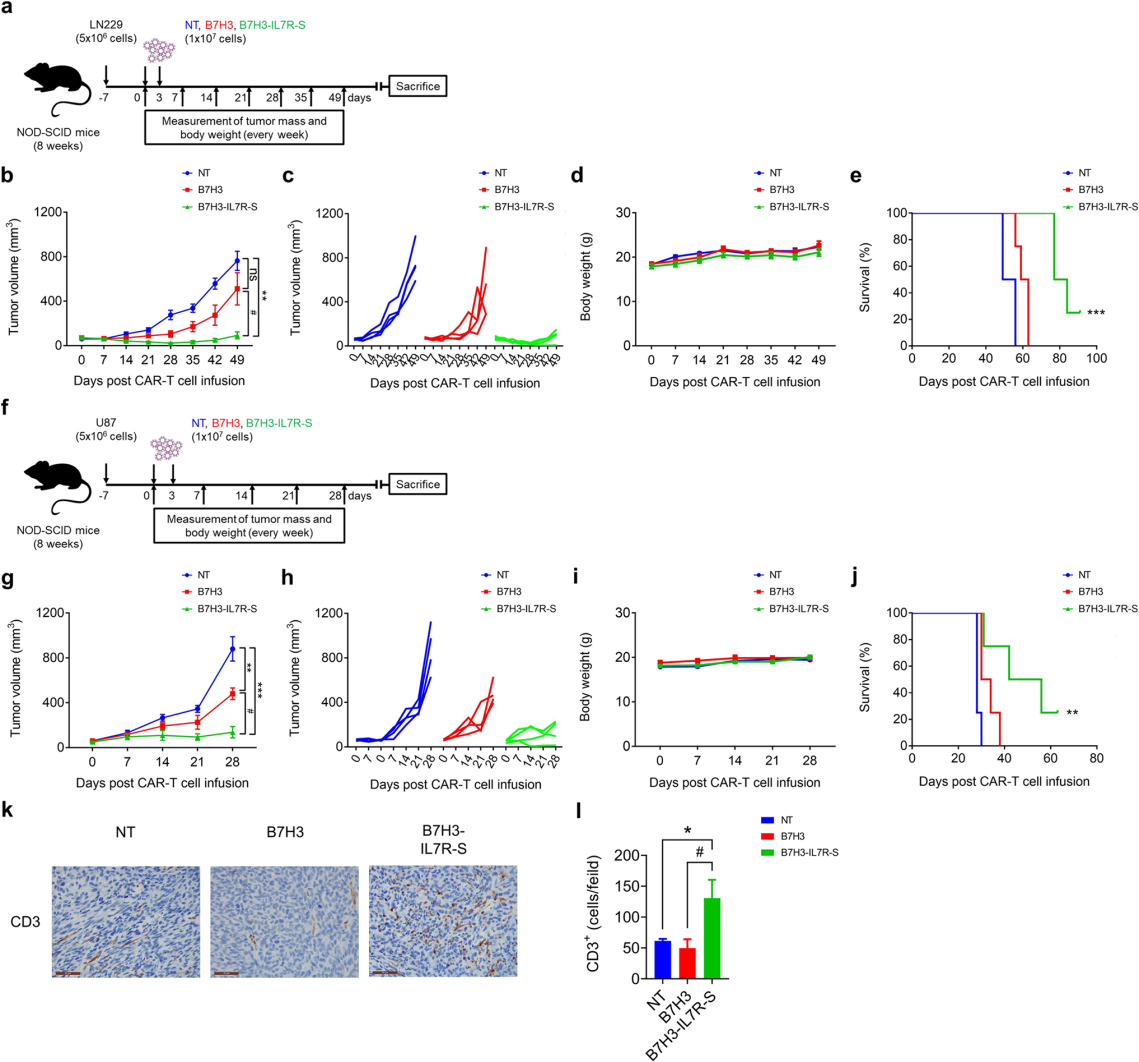
Antitumor responses of B7H3 IL7R-S CAR-T cells in xenograft mouse models of GBM. **a** NOD/SCID mice bearing LN229 glioblastoma (5 × 10^6^) were treated with B7H3, B7H3-IL7R-S CAR-T or NT cells (1 × 10^7^) for 2 doses at 3-day intervals. Tumor volume was measured every week using a caliper. **b** The mean tumor volume and **c** individual tumor volumes are shown. The data are shown as the mean ± S.E.M. (*n* = 4) ns = not significant, ^**^P < 0.01 versus NT control group, ^#^*P* < 0.05 versus B7H3-treated group (one-way ANOVA). **d** The body weights of mice bearing LN229 tumor cells were monitored before and after the infusion of CAR-T cells. **e** Kaplan–Meier survival curves over time in each group (*n* = 4) ^***^P < 0.01 versus B7H3-treated group. **f** Schematic representation of the experimental protocol using U87 cell-derived xenografts. **g** Mean tumor volume and **h** individual tumor volumes are shown. The data are shown as the mean ± S.E.M. (*n* = 4). ^**^P < 0.01; ^***^P < 0.001 versus NT control group; ^#^*P* < 0.05 versus B7H3-treated group (one-way ANOVA). **i** The body weight of mice bearing U87 tumor cells. **j** Kaplan–Meier survival curves over time in each group. ^**^P < 0.01 versus B7H3-treated group. **k** Representative IHC image of human CD3 staining in tumor tissues from LN229 tumor-bearing mice on day 49. All images were obtained at 10 × magnification; the scale bar represents 50 μm. **l** Quantification of CD3-positive cells that infiltrated into tumors was performed with ImageJ software. The data are shown as the mean ± S.E.M. (n = 3). ^*^P < 0.05 versus NT; ^#^*P* < 0.05 versus B7H3-treated group (one-way ANOVA)

As B7H3-IL7R-S exhibited superior antitumor activity compared with that of other intrinsic IL7Rα constructs, we tested the antitumor effects of B7H3-IL7R-S in comparison with those of conventional B7H3 CAR-T cells in xenograft models of glioblastoma. NOD/SCID mice were engrafted subcutaneously with two different GBM cell lines, as illustrated in Fig. 6a, f. B7H3-IL7R-S CAR-T cells significantly promoted tumor eradication and prolonged survival compared to B7H3 CAR-T cells in LN229 tumor-bearing mice (Kaplan − Meier, log-rank tests and Cox regression analyses were used; *P* < 0.001) (Fig. 6b–c, e). No difference in the weight of the mice was observed among the three groups (Fig. 6d). The antitumor efficacy of B7H3 CAR-T cells was also confirmed in mice bearing highly invasive U87 tumor xenografts (Fig. 6f). Similarly, the administration of B7H3-IL7R-S CAR-T cells resulted in potent antitumor activity, with a growth inhibition rate of 84.4% (Fig. 6g − h). In addition, as indicated in Fig. 6j, a trend toward longer disease-free survival was observed in mice treated with B7H3-IL7R-S CAR-T cells (Kaplan–Meier, log-rank tests and Cox regression analyses were used; *P* < 0.01), and no difference in the weight of the mice was observed among the three experimental groups (Fig. 6i). In addition, immunohistochemistry (IHC) of tumor biopsies obtained from mice bearing LN229 cells demonstrated greater CD3^+^ T-cell infiltration in the B7H3-IL7R-S group than in the B7H3 and NT groups (Fig. 6k–l). These results demonstrated the enhanced antitumor activity of B7H3-IL7R-S CAR-T cells in xenograft mouse models.

## Discussion

We constructed B7H3-targeting CAR structures composed of three different lengths of the IL7Rα signaling domain and assessed their antitumor activities against glioblastoma in vitro and in vivo. Our study revealed the beneficial role of IL7 receptor signaling in the antitumor activity of B7H3 CAR-T cells against glioblastoma. Our results demonstrated that B7H3 IL7R-S CAR-T cells can maintain their antitumor activity under repetitive antigen stimulation. In an in vitro study, these B7H3 IL7R-S CAR-T cells demonstrated sustained activation of the transcription factor STAT5, reduced activation-induced cell death and increased T-cell persistence. Additionally, we demonstrated the superior antitumor activity of B7H3 IL7R-S CAR-T cells in vivo compared to conventional second-generation B7H3 CAR-T cells in two distinct glioblastoma-bearing xenograft mouse models. Taken together, our findings suggest that incorporating IL7R-S represents a promising strategy for enhancing the efficacy of CAR-T cells in the treatment of solid tumors.

Resistance to CAR-T therapy often results from inadequate T-cell persistence, leading to poor tumor response and increased relapse rates [6, 7]. CAR-T cells with a naïve or central memory phenotype exhibit increased proliferation potential, longevity, and functionality, leading to enhanced antitumor activity and prolonged persistence [33, 34]. Interleukin-7 (IL-7) has been demonstrated to improve CAR-T cell proliferation and effector functions while preventing exhaustion [12] and maintaining a less differentiated state [35, 36]. The downstream signaling of IL7R plays a crucial role in promoting T-cell survival through the activation of STAT5 [37]. Our B7H3 IL7R-S CAR contains the 8-amino acid Box1 motif and the conserved tyrosine residue Y449 derived from the IL7Rα cytoplasmic domain. The conserved Box1 motif of class I cytokine receptors, including IL7Rα, is required for JAK kinase association [38, 39]. Activated JAK1 then phosphorylates Y449, regulating the antiapoptotic gene Bcl2 in murine T cells [40]. In addition, Y449 in the IL7Rα cytoplasmic domain has been identified as a crucial docking site for PI-3 K and STAT5 and is essential for the survival signal induced by IL7 signaling [41].

In our study, we observed that B7H3 IL7R-S CAR-T cells maintained the phosphorylation of STAT5 (pSTAT5) more effectively than did the other IL7R constructs, which correlated with the superior antitumor activity of IL7R-S in the tumor rechallenge assay. RNA sequencing revealed that incorporation of IL7R in the S construct led to the upregulation of several genes associated with T-cell proliferation. One noteworthy gene is an SRC family kinase, FYN. A recent study showed that FYN phosphorylates CAR ITAMs, thereby promoting the proliferation and survival of CAR-T cells harboring the CD28 costimulatory domain [42]. Additionally, the incorporation of IL7R signaling in our CAR construct effectively mitigated activation-induced cell death, which correlated with the downregulation of proapoptotic genes and the upregulation of antiapoptotic genes.

Interestingly, we observed a decrease in IFN-γ and IL-2 production following antigen stimulation in B7H3 CAR-T cells harboring IL7Rα. We speculate that this may be attributed to the higher population of effector cells in the conventional B7H3 group. However, this could be an advantage, as this reduced secretion of IFN-γ, without compromising antitumor activity, may contribute to a decreased incidence of cytokine release syndrome (CRS) [43, 44]. In this study, we also noted that, compared to the conventional B7H3 CAR construct, B7H3-IL7R CAR-T cells secrete higher levels of IL-10 upon antigen stimulation. The cytokine secretion results were consistent with the upregulation of the IL-10 gene according to the RNA sequencing data. To our knowledge, the mechanism by which IL-7 signaling activation leads to the production of IL-10 has not been validated. However, previous studies have reported increased IL-10 levels in the serum of chronically virus-infected mice treated with recombinant IL-7 [45]. Additionally, codelivery of IL-7 in a DNA plasmid model resulted in the induction of antigen-specific CTLs and increased production of the Th2 cytokine IL-10 [46]. More recent studies have unveiled a new role for IL-10 in adoptive T-cell therapy. Through the enhancement of mitochondrial oxidative phosphorylation, IL-10 induces the expansion and effector function of exhausted tumor-infiltrating T cells [47]. Furthermore, engineering CAR-T cells to secrete IL-10 enhances their proliferation and effector function, leading to durable eradication of solid tumors [48].

Previous studies have demonstrated that enhancing CAR-T-cell efficacy through the addition of interleukin-7 receptor (IL7R) signaling, by incorporating IL-7 secretion, expression of constitutively activated IL7R, or inverted cytokine receptor IL-4 to IL-7 can be an effective strategy for enhancing CAR-T cell function and restoring antitumor activity in the immunosuppressive tumor microenvironment [13–16, 35, 49]. Our innovative IL7R-S construct provides further evidence of the advantages of IL7R signaling and underscores the importance of cytokine signaling in CAR-T cell function, particularly in solid tumors. Our approach also offers an alternative system that harnesses the benefits of IL7R signaling within a single CAR construct. The improved efficacy of IL7R-S compared to that of 2nd-generation B7H3 CAR-T cells was clearly demonstrated in in vivo experiments involving glioblastoma. However, further investigations in a more relevant orthotropic model of glioblastoma and an extension of the study to other solid tumors, considering that B7H3 is widely expressed in multiple cancers, will be necessary to assess the clinical potential of our IL7R-S. In addition, assessing the integration site status of our IL7R-S construct is essential to ensure its safety before clinical translation. This analysis could help identify potential genomic alterations and associated risks, such as insertional mutagenesis, thereby enhancing the safety profile of the therapeutic intervention.

In conclusion, our cellular and animal experiments showed that B7H3 CAR-T cells combined with IL7R exhibited noteworthy antitumor activity against GBM, surpassing that of second-generation B7H3 CAR-T cells. The incorporation of IL7Rα signaling leads to sustained pSTAT5 expression, a reduction in proapoptotic genes and a reduction in activation-induced cell death. Therefore, our findings indicate that B7H3 IL7R-S CAR-T cells have the potential to serve as a promising treatment strategy for GBM.

### Supplementary Information

Below is the link to the electronic supplementary material.

Supplementary file1 (DOCX 1161 KB)

## Data Availability

The datasets generated and/or analyzed during the current study are available from the corresponding author upon reasonable request.

## Abbreviations

B7H3: B7 homolog 3 protein (also known as CD276)
CAR: Chimeric antigen receptor
CBA: Cytometric bead array
CD3: Cluster of differentiation 3
CD25: Cluster of differentiation 25
CD69: Cluster of differentiation 69
CRS: Cytokine release syndrome
DEGs: Differentially expressed genes
FACS: Fluorescence activated cell sorting
GBM: Glioblastoma
GO: Gene Ontology
GvHD: Graft-versus-host disease
i.v.: Intravenous
IFN-γ: Interferon-gamma
IgG2: Immunoglobulin G2
IL-2: Interleukin-2
IL-7: Interleukin-7
IL-15: Interleukin-15
IL7R: Interleukin-7 receptor
MOI: Multiplicity of infection
NOD-SCID: NOD/ShiJic-scid Jcl
PBMC: Peripheral blood mononuclear cell
PD1: Programmed cell death protein 1
RNA-seq: RNA-sequencing
s.c.: Subcutaneous
STAT5: Signal transducer and activator of transcription 5
TCR: T-cell receptor
TN: Naïve T cells
TNF-α: Tumor necrosis factor-alpha
TSCM: Stem-like memory T cells
41BB: Costimulatory molecule (also known as CD137 or TNFRSF9)
