# Supplementary material for: Incorporating IL7 receptor alpha signaling in the endodomain of B7H3-targeting chimeric antigen receptor T cells mediates antitumor activity in glioblastoma
Source: Cancer Immunol Immunother. 2024 Apr 15;73(6):98. doi: 10.1007/s00262-024-03685-7 (PMC11018726; doi:10.1007/s00262-024-03685-7)
Supplement: Supplementary file 1 — Supplementary file1 (DOCX 1161 KB) [file 262_2024_3685_MOESM1_ESM.docx]

Cancer Immunology, Immunotherapy (submitted in 2023) - Nithidol Sakunrangsit et al.


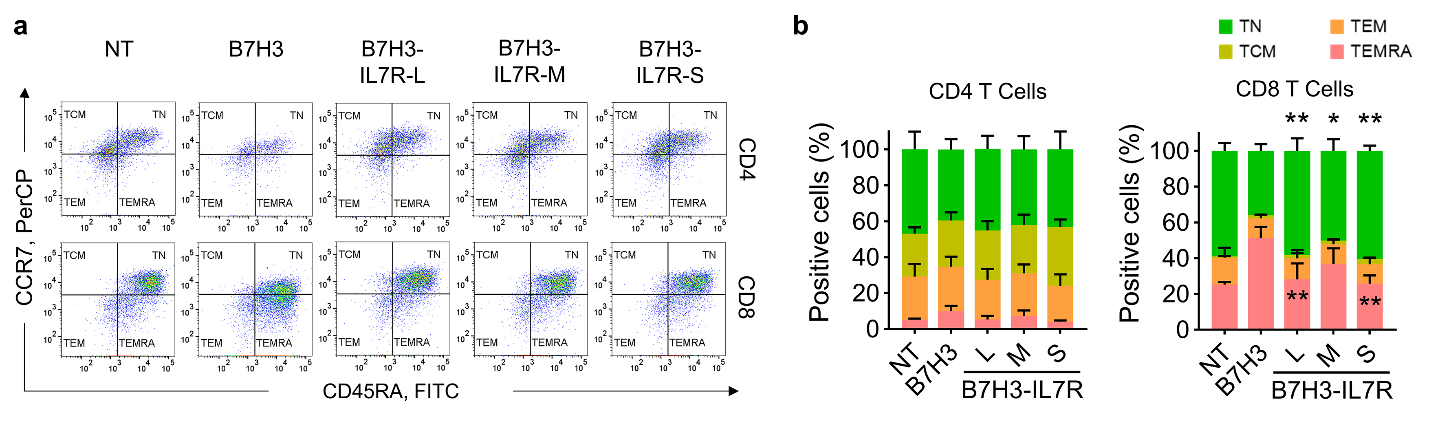


**Supplementary Figure 1** Characterization of the immune phenotype of CD4+ and CD8+ T-cell subpopulations. **a** Flow cytometry analysis of the memory T-cell subpopulations identified as follows: naïve T cells (TN, CD45RA+CCR7+), central memory T cells (TCM, CD45RA‒CCR7+), effector memory T cells (TEM, CD45RA‒CCR7‒), and effector T cells (TEMRA, CD45RA+CCR7‒) in CAR-T-cell products on day 11 after transduction. **b** The percentage of positive cells for each phenotype in the CD4+ and CD8+ T cell populations. Data are presented as the mean ± S.E.M. (*n*=3). * P < 0.05, ** P < 0.01 vs. B7H3 CAR-T cells (two-way ANOVA).

**
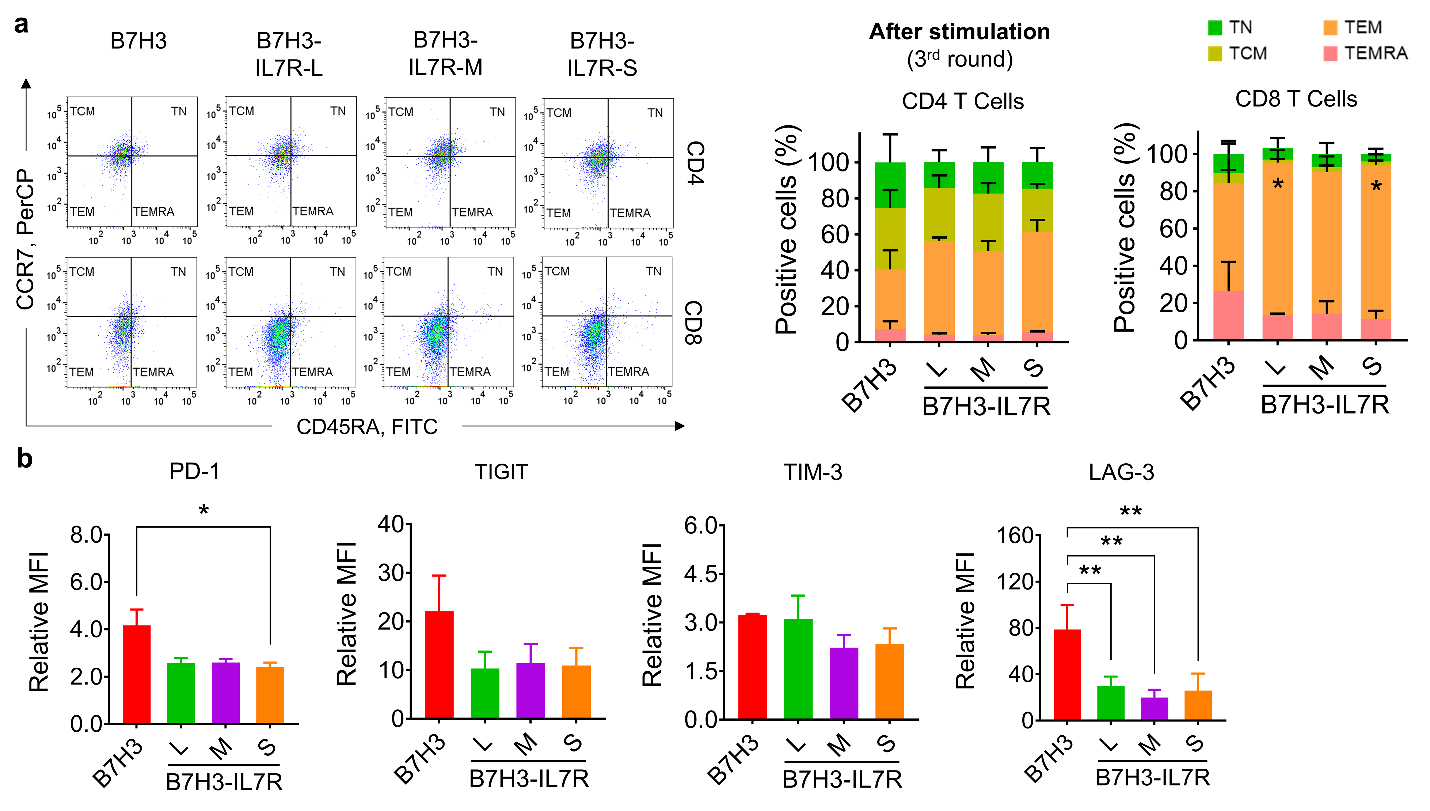
 Supplementary Figure 2** Immune phenotype of CAR T cells after the third re-exposure with U87 cell in tumor rechallenge assay. **a** Flow cytometry analysis of the memory T-cell subpopulations identified as follows: naïve T cells (TN, CD45RA+CCR7+), central memory T cells (TCM, CD45RA‒CCR7+), effector memory T cells (TEM, CD45RA‒CCR7‒), and effector T cells (TEMRA, CD45RA+CCR7‒). Bar graph presents the percent positive cells of phenotype in the CD4+ and CD8+ T-cell subpopulations. Data are shown as the mean ± S.E.M. (*n*=3) ^*^ P < 0.05 TEM of B7H3-L and B7H3-S CAR-T cells vs. B7H3 CAR-T cells (two-way ANOVA). **b** Expression of the exhaustion markers including PD-1, TIGIT, TIM-3, LAG-3 on CAR-T cells after the third re-exposure with U87. Data are shown as the mean ± S.E.M. (*n*=3). ^*^ P < 0.05, ^**^ P < 0.01 (one-way ANOVA).

**
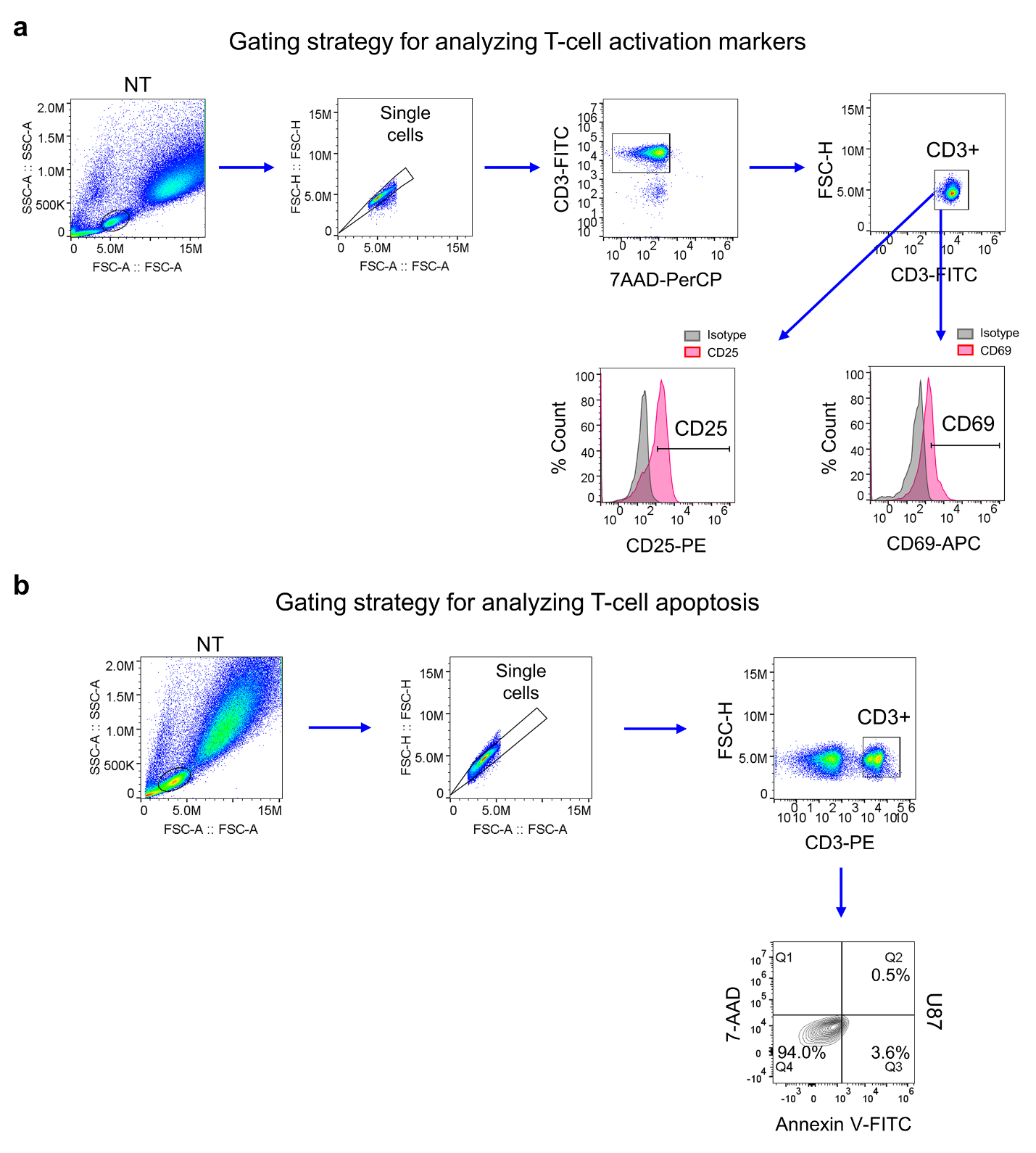
 Supplementary Figure 3** Gating strategies for analyzing T cells **a** Activation markers, including CD25 and CD69, on CD3+ T cells were gated after stimulation with target cells for 72 hours. Dead cells were excluded using a fluorescent cell viability dye (7AAD). **b** After stimulation with target cells, CD3+ T cells were gated, and the apoptotic population was determined using 7AAD and Annexin V.
